# Social Experience Is Sufficient to Modulate Sleep Need of *Drosophila* without Increasing Wakefulness

**DOI:** 10.1371/journal.pone.0150596

**Published:** 2016-03-03

**Authors:** Shahnaz Rahman Lone, Sheetal Potdar, Manishi Srivastava, Vijay Kumar Sharma

**Affiliations:** 1 Chronobiology Laboratory, Evolutionary and Organismal Biology Unit, Jawaharlal Nehru Centre for Advanced Scientific Research, Jakkur, Bangalore, Karnataka, India; 2 Behavioural Neurogenetics Laboratory, Evolutionary and Organismal Biology Unit, Jawaharlal Nehru Centre for Advanced Scientific Research, Jakkur, Bangalore, Karnataka, India; Wake Forest University, UNITED STATES

## Abstract

Organisms quickly learn about their surroundings and display synaptic plasticity which is thought to be critical for their survival. For example, fruit flies *Drosophila melanogaster* exposed to highly enriched social environment are found to show increased synaptic connections and a corresponding increase in sleep. Here we asked if social environment comprising a pair of same-sex individuals could enhance sleep in the participating individuals. To study this, we maintained individuals of *D*. *melanogaster* in same-sex pairs for a period of 1 to 4 days, and after separation, monitored sleep of the previously socialized and solitary individuals under similar conditions. Males maintained in pairs for 3 or more days were found to sleep significantly more during daytime and showed a tendency to fall asleep sooner as compared to solitary controls (both measures together are henceforth referred to as “sleep-enhancement”). This sleep phenotype is not strain-specific as it is observed in males from three different “wild type” strains of *D*. *melanogaster*. Previous studies on social interaction mediated sleep-enhancement presumed ‘waking experience’ during the interaction to be the primary underlying cause; however, we found sleep-enhancement to occur without any significant increase in wakefulness. Furthermore, while sleep-enhancement due to group-wise social interaction requires Pigment Dispersing Factor (PDF) positive neurons; PDF positive and CRYPTOCHROME (CRY) positive circadian clock neurons and the core circadian clock genes are not required for sleep-enhancement to occur when males interact in pairs. Pair-wise social interaction mediated sleep-enhancement requires dopamine and olfactory signaling, while visual and gustatory signaling systems seem to be dispensable. These results suggest that socialization alone (without any change in wakefulness) is sufficient to cause sleep-enhancement in fruit fly *D*. *melanogaster* males, and that its neuronal control is context-specific.

## Introduction

Sleep-like state is widely observed across animal kingdom, from simple nematodes to complex mammals; yet its advantages are not entirely clear [1]. Sleep is believed to help in clearing toxic waste build-up in neurons to safeguard organisms from its deleterious effects [2], and sleep deprivation severely affects lifespan [3–5]. Sleep plays a critical role in maintaining synaptic plasticity and in reducing energy cost [6]. For instance, synaptic strength is higher when animals are awake and it drops down to sustainable levels during sleep, perhaps to prepare them for renewed challenges of the next cycle. In mammals as well as in *Drosophila*, sleep after a training session is essential for memory consolidation through synaptic potentiation [7–9]. In contextual fear conditioning in mice, sleep deprivation after a training session results in impaired memory consolidation [7]. Also in *Drosophila*, sleep helps in long term memory (LTM) formation through mass training, which under normal circumstances is not known to induce LTM [9]. Furthermore, according to the ontogenetic theory of sleep, sleep during the early developmental stages is critical for normal patterning of the brain [10]. Flies, like many other organisms, sleep more during the early life stages, which is believed to help certain parts of their brain such as VAIv glomeruli to grow, and sleep deprivation during these stages results in impaired development of the VAIv glomeruli [11].

Sleep in fruit flies *D*. *melanogaster* is defined as sustained periods of immobility, increased arousal threshold, altered electrical activity of the brain and homeostatic regulation [12–14]. Sleep depends on a number of factors including age, sex and environmental conditions [15]. Social environment of an organism is also known to have a positive but short-lived effect on daytime sleep, which is considered to be adaptive, at least for organisms that normally experience a more enriched social environment during the day as compared to night.

*Drosophila* is known to be sensitive to its social environment and this sensitivity is seen during both larval as well as adult stages. When flies are maintained as larvae in high density cultures, the number of kenyon cells and volume of mushroom body calyx increase considerably [16], and adults that emerge out of such cultures sleep significantly more as compared to those that are maintained in low density cultures [16, 17]. Similarly, flies maintained as adults in same-sex or mixed-sex social groups are found to sleep significantly more after they are separated as compared to solitary controls [18, 19]. It has also been observed that *Drosophila* responds to social cues by increasing the number of synapses in the brain involved in processing such information [16], which suggests a link between social signals and synaptic plasticity. Notwithstanding the importance of synaptic plasticity for critical behaviours such as sleep, it is surprising to note that thus far there have been only a few studies that examined the effect of social environment on sleep [18, 19]. In a couple of elegant studies it was shown that ‘waking experience’ in socially interacting flies (in either same-sex or mixed-sex groups of *n* = 30–40 individuals), causes sleep-enhancement after the interaction [18, 19]. It was proposed that social cues activate the arousal promoting large ventral lateral neurons (I-LNvs) via visual and olfactory systems, which in turn cause synaptic potentiation leading to enhanced sleep [19, 20]. As a striking deviation from this we report that in *D*. *melanogaster*, social interaction between as few as two males is sufficient to cause sleep-enhancement independent of waking experience. To this end, we designed experiments where we formed same-sex (male or female) pairs, and maintained them for a period ranging from 1 to 4 days in glass tubes (7 mm × 65 mm), and used solitary individuals of the respective sex, maintained in similar conditions, as controls. While females did not show any detectable effect of social interaction on sleep, socialized males slept significantly more during daytime and displayed tendency to fall asleep sooner as compared to solitary controls. This sleep phenotype is strain independent and does not require the previously implicated arousal promoting I-LNv neurons for its persistence but uses dopamine and olfactory signaling, while visual and gustatory signaling systems appear to be dispensable.

## Materials and Methods

*Canton S (CS)*, *Oregon R (OR)*, *Iso31*, *norpA*, *Gr5aGAL4*, *Gr66aGAL4*, *GR33aGAL4*, *Orco*, *per*^0^, *tim*^0^, *Clk*^jrk^, *Or83bGAL4*, *pleGAL4*, *cry(39)GAL4*, *PdfGAL4*, *UAShid*, *UASKir2*.*1*, *UASdORKC1*, *UASshibire*^*ts*^ and *UAStnt* (active and inactive) fly lines were obtained from the laboratory of Todd Holmes, National Centre for Biological Sciences and Bloomington Stock Centre. Flies were raised on corn medium and were separated as virgins immediately after emergence. Freshly emerged flies were maintained in same-sex groups of 30–40 individuals until they were 4 days old. Four day old males were divided into two groups; males from the first group were paired and introduced into 7 mm × 65 mm glass tubes with corn medium at one end and cotton plug at the other, whereas males from the second group were kept solitarily in similar tubes. Carbon dioxide was used only for a brief period of time to load flies into the activity tubes. Similarly, females were also paired or kept solitarily. Flies were maintained in pairs for a period of 1, or 2, or 3, or 4 days, after which they were separated without using anesthesia, by transferring them into 5 mm × 65 mm glass tubes for recording locomotor activity using *Drosophila* activity monitors (Trikinetics, Waltham, USA). Flies maintained solitarily in 7 mm × 65 mm glass tubes for a period of 4 days were also transferred into 5 mm × 65 mm glass tubes for recording locomotor activity. Average sleep of flies over 2–3 days after separation was used for analysis. For monitoring activity of flies during the social interaction, paired flies (males or females) and their solitary controls were introduced into 7 mm × 65 mm glass tubes.

Sleep was estimated as 5 min or more of continuous inactivity as defined previously [12, 13]. Sleep latency in minutes was estimated as the time duration until the first sleep episode after lights-ON (daytime latency) or lights-OFF (nighttime latency), and sleep was calculated using custom made software [21]. Change in sleep (Δsleep) was estimated by subtracting the mean daytime or nighttime sleep of solitary controls from daytime or nighttime sleep of each socialized fly [18]. We performed Analysis of Variance (ANOVA) followed by post hoc multiple comparisons using Tukey’s honest significant difference (HSD) test, or Student’s *t*-test with Bonferroni correction, depending on the data sets (see results for details). Error bars in the figures are standard error around means (SEM). All our statistical analyses were implemented on Statistica (Statsoft, 1995).

## Results

### Pair-wise interaction among males results in sleep-enhancement

**Fig 1:**
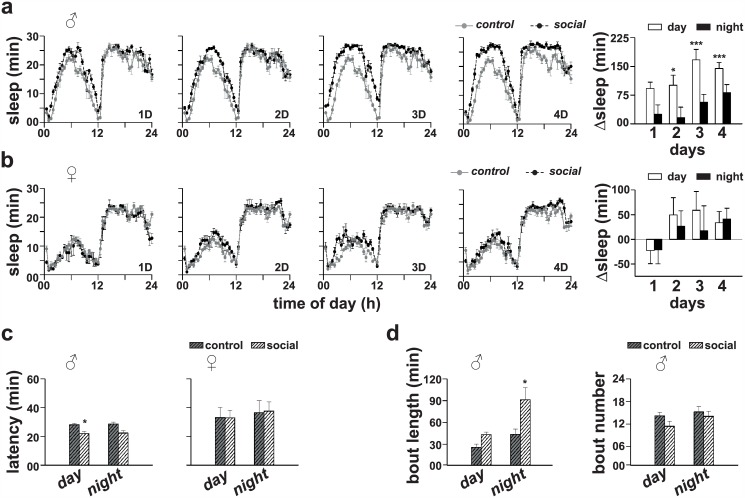
Sleep-enhancement in males due to pair-wise social interaction. (a, left) Sleep profiles of *Canton S* (*CS*) males following pair-wise social interaction with other males for (1D) 1 day, (2D) 2 days, (3D) 3 days or (4D) 4 days. In the sleep profiles, black circles and dark broken lines represent sleep of flies subjected to pair-wise social interaction, whereas grey circles and grey solid lines represent sleep of solitary controls. (a, right) Bar graphs show change in sleep of socialized males as compared to solitary controls and there is an increase in daytime sleep (white bars) in socialized males as compared to solitary controls (*p* = 0.09 for 1D, *p* = 0.06 for 2D, *p <* 0.0005 for 3D and 4D, ANOVA followed by post hoc multiple comparison by Tukey’s test). Nighttime sleep (dark bars) of socialized males does not differ from that of solitary controls. (b) Sleep profiles of *CS* females following pair-wise social interaction with other females for (1D) 1 day, (2D) 2 days, (3D) 3 days or (4D) 4 days. Both daytime and nighttime sleep does not differ between socialized and solitary control females (*p* > 0.05, ANOVA followed by post hoc by Tukey’s test). (c) Following 4 days of social interaction, daytime sleep latency of males is significantly reduced (*p <* 0.01, Student’s *t*-test) as compared to solitary controls. Although nighttime sleep latency also shows a similar decrease, it did not reach statistically significant levels (*p* > 0.05, Student’s *t*-test). In case of females, both daytime and nighttime sleep latency does not differ between socialized and solitary individuals. (d) While there is a statistically significant increase in sleep bout length during nighttime (*p* < 0.05, Student’s *t*-test) in socialized males as compared to solitary controls, bout number does not different significantly among socialized and solitary control males. Data is presented as mean ± SEM (standard error of means) and *n* = 16 for each group of males and females. Asterisks over each bar indicate statistically significant difference between socialized and solitary control flies unless mentioned otherwise, where *p <* 0.05 is represented by single asterisk, *p <* 0.005 by two asterisks and *p <* 0.0005 by three asterisks. Other details about the bar graphs are same as in 1a.

*CS* flies were maintained as male–male or female–female pairs in glass tubes for a period ranging from 1 to 4 days, following which they were separated by transferring individually into fresh glass tubes without anesthesia to record their locomotor activity behaviour. Sleep was estimated from the activity data as 5 min or more of continuous inactivity. ANOVA showed a statistically significant effect of time (day/night) of day (*p* < 0.0001) and number of days of socialization (*p* < 0.0001). Although males showed an increase in daytime sleep after 1 or 2 day(s) of social interaction, it did not reach statistically significant levels (*p* = 0.09 for 1 day and *p* = 0.06 for 2 days, post hoc multiple comparisons using Tukey’s test; Fig 1a). Following 3 or 4 days of socialization, males showed a statistically significant increase in daytime sleep as compared to solitary controls (*p* < 0.0005, post hoc multiple comparisons using Tukey’s test; Fig 1a), however, there was no statistically significant difference (*p* > 0.05) in daytime sleep when compared across different days of socialization. These results suggest that 3 days of pair-wise interaction is enough to cause sleep-enhancement in males. Nighttime sleep of males did not change significantly even after 4 days of socialization (*p* > 0.05, post hoc multiple comparisons using Tukey’s test; Fig 1a). Analysis revealed that daytime sleep latency in socialized males decreased significantly as compared to solitary controls (*p* < 0.01, *t*-test; Fig 1c). Although nighttime sleep latency was also reduced, it did not reach statistically significant levels (*p* > 0.05, *t*-test; Fig 1c). Females on the other hand did not show any statistically significant effect of socialization on daytime or nighttime sleep (*p* > 0.05, ANOVA followed by post hoc comparisons by Tukey’s test; Fig 1b), or on sleep latency (*p* > 0.05, *t*-test; Fig 1c). We analyzed effect of socialization on the number of sleep bouts and bout duration, only in males. Although bout number (*p* > 0.05 during day as well as for nighttime sleep, *t*-test; Fig 1d) did not change significantly in socialized males, bout duration was significantly increased (*p* < 0.05, *t*-test) during nighttime but remained unchanged during daytime (*p* > 0.05, *t*-test; Fig 1d). These results suggest that sleep in males is sensitive to previously experienced social cues and that it is significantly increased following 3 or more days of pair-wise interaction with other males. Therefore, for the rest of our study we have used 4 days of pair-wise interaction among males as a standard protocol to assess sleep-enhancement.

### Pairing of flies does not affect their sleep during the social interaction

**Fig 2:**
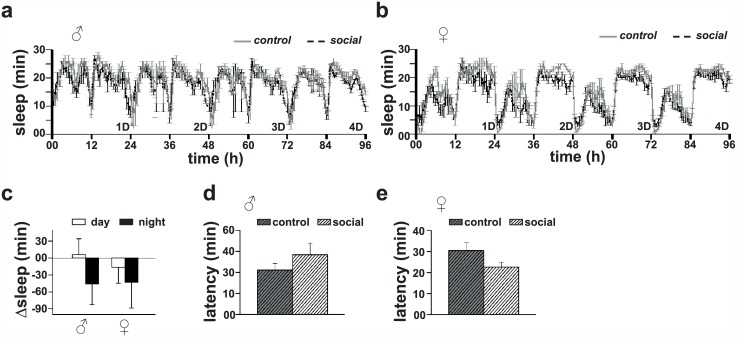
Sleep in males during social interaction. (a) Sleep profiles of males across 4 days of social interaction. (b) Sleep profiles of females across 4 days of social interaction. (c) Change in sleep bars (mean ± SEM) for males (*n* = 16 and 24, for socialized and control groups respectively) and females (*n* = 14 and 22, for socialized and control groups respectively), where white and dark bars represent sleep during day as well as nighttime. Sleep of socially interacting males is not significantly different (*p* = 0.99 for daytime and *p* = 0.50 for nighttime, Student’s *t*-test) from that of solitary controls. Sleep of socially interacting females is also not significantly different (*p* = 0.98 for daytime and *p* = 0.76 for nighttime, Student’s *t*-test) from that of solitary controls. (d, e) Sleep latency of socially interacting (d) males and (e) females is comparable (*p >* 0.05, Student’s *t*-test) to that of solitary controls. Other details are same as in Fig 1.

Since previous studies on socialization mediated sleep-enhancement were performed on flies maintained in groups, the effect of socialization on sleep during the interaction could not be assessed, due to difficulties in simultaneously estimating sleep of a large number of individuals. In the present study, since we have used a social group of only two individuals, we decided to monitor sleep of the interacting pairs during the social interaction in slightly larger activity tubes (7 mm × 65 mm glass tubes) as compared to the ones normally used for recording locomotor activity of solitary individuals (5 mm × 65 mm glass tubes). ANOVA on sleep levels revealed that socialization does not have any statistically significant effect on amount of sleep during the interaction (*p* = 0.39). Sleep in socially interacting males did not differ as compared to solitary controls (*p* = 0.99 for daytime sleep and *p* = 0.50 for nighttime sleep; Fig 2a and 2c). This was true also for females (*p* = 0.98 for daytime sleep and *p* = 0.76 for nighttime sleep; Fig 2b and 2c). Analysis on sleep latency revealed that socialization does not have any statistically significant effect on sleep latency of male (Fig 2d) or female (Fig 2e) flies during the interaction (*p* > 0.05, *t*-test). These results suggest that sleep-enhancement observed after the separation of flies following socialization (Fig 1) is not a rebound response to sleep loss during the interaction but due to the modulation of sleep need [18, 19].

### Sleep-enhancement is not strain-specific

**Fig 3:**
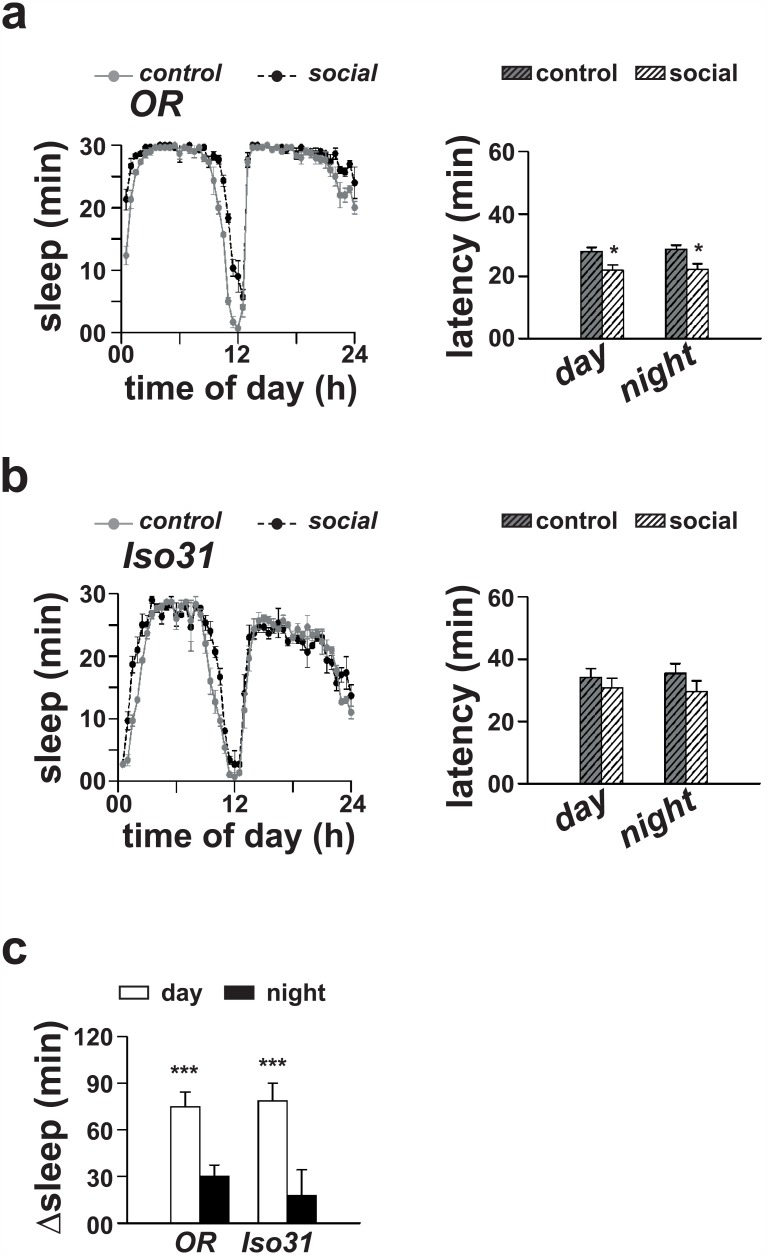
Sleep-enhancement in two other strains of *D*. *melanogaster*. (a, left) Sleep profiles of *Oregon R* (*OR*) males following 4 days of pair-wise social interaction. (a, right) Daytime and nighttime sleep latency is significantly lower (*p <* 0.05, Student’s *t*-test) as compared to that of solitary controls. (b, left) Sleep profiles of *Iso31* males following 4 days of pair-wise social interaction. (b, right) Although sleep latency of socialized males is also decreased, it did not reach statistically significant levels (*p >* 0.05, Student’s *t*-test). (c) Daytime sleep of socialized *OR* males (*p <* 0.0001, Student’s *t*-test; *n* = 16 for each group) and *Iso31* males (*p <* 0.0005, Student’s *t*-test; *n* = 15 for each group) is significantly greater as compared to that of solitary controls. Other details are same as in Fig 1.

To examine if social interaction mediated sleep-enhancement in males is also observed in other “wild type” strains of *Drosophila*, we studied two additional fly lines—*OR* and *Iso31*. Student’s *t*-test with Bonferroni correction revealed a statistically significant effect of socialization on daytime sleep in *OR* strain (*p* < 0.0001; Fig 3a and 3c); however, their nighttime sleep did not change significantly (*p >* 0.05; Fig 3a and 3c). Socialization also had a statistically significant effect on day as well as nighttime sleep latency (*p* < 0.05, *t*-test; Fig 3a). Similar to *OR* flies, socialization also had a statistically significant effect on daytime sleep of *Iso31* males (*p <* 0.0005, *t*-test; Fig 3b and 3c), while their nighttime sleep remained unaltered (*p* > 0.05, *t*-test). Although a trend of decreased day as well as nighttime sleep latency was also seen, it did not reach statistically significant levels (*p >* 0.05, *t*-test; Fig 3b). These results suggest that social interaction mediated sleep-enhancement is not strain-specific.

### Olfactory signals mediate sleep-enhancement

To examine the sensory signals involved in sleep-enhancement, we blocked different modes of sensory perception either using mutations affecting functions of the involved genes, or by causing ablation or silencing of the sensory neurons.

**Fig 4:**
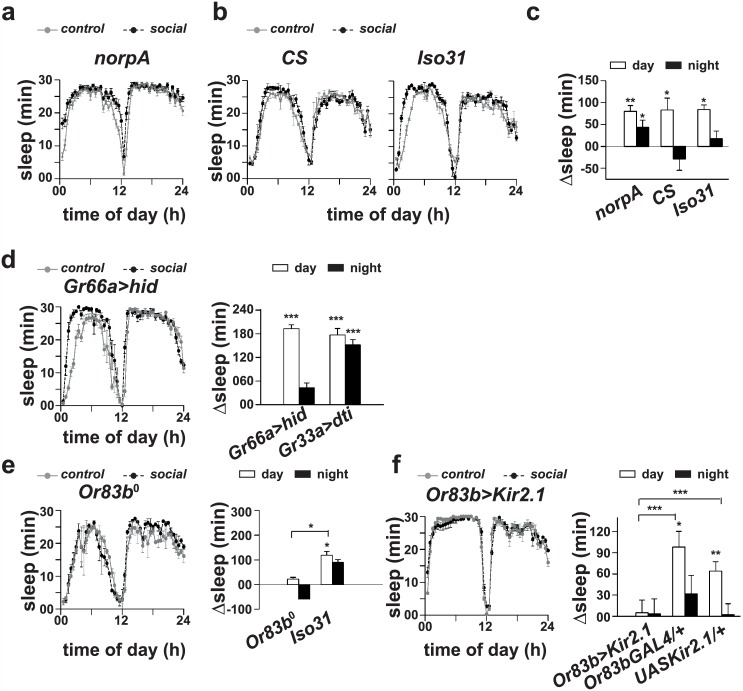
Olfactory cues mediate sleep-enhancement. (a) Sleep profiles of *norpA* males following 4 days of pair-wise social interaction in 12:12 h light/dark cycles (LD12:12). (b) Sleep profiles of *CS* and *Iso31* males following pair-wise social interaction for 4 days in constant darkness (DD). (c) Sleep analysis revealed that daytime sleep is significantly increased in socialized *norpA* males as compared to solitary controls (*p <* 0.0005 for daytime and *p <* 0.02 for nighttime, Student’s *t*-test; *n* = 21 and 24 for socialized and control groups respectively). Daytime sleep of socialized *CS* (*p <* 0.02, Student’s *t*-test; *n* = 14 and 16 for socialized and control groups respectively) and *Iso31* (*p <* 0.01, Student’s *t*-test; *n* = 29 and 18 for socialized and control groups respectively) males is also significantly increased as compared to that of solitary controls. (d, left) Sleep profiles and (d, right) change in sleep of *Gr66aGAL4>UAShid* and *Gr33aGAL4>UASdti* males following 4 days of pair-wise social interaction. Daytime sleep of socialized *Gr66aGAL4>UAShid* males (*p <* 0.0001, Student’s *t*-test; *n* = 16 and 14 for socialized and control groups respectively) and socialized *Gr33aGAL4>UASdti* males (*p <* 0.0001, Student’s *t*-test; *n* = 32 and 19 for socialized and control groups respectively) is significantly increased in comparison to that of solitary controls. Nighttime sleep of *Gr33aGAL4>UASdti* males is also significantly increased (*p <* 0.0005, Student’s *t*-test) in comparison to that of that of solitary controls. (e, left) Sleep profiles and (e, right) change in sleep of *Orco* males following 4 days of pair-wise social interaction. Sleep analysis revealed that day as well as nighttime sleep of socialized *Orco* males is comparable (*p >* 0.05; *n* = 27 and 20 for socialized and control groups respectively) to solitary controls, whereas daytime sleep of socialized *Iso31* males is significantly greater as compared to that of solitary controls (*p <* 0.05; *n* = 13 and 10 for socialized and control groups respectively), daytime sleep-enhancement in *Iso31* flies is significantly greater (*p <* 0.05) than that of *Orco* flies. (f, left) Sleep profiles and (f, right) change in sleep of *Or83bGAL4>UASKir2*.*1* males following 4 days of pair-wise social interaction. (f, right) Day as well as nighttime sleep of socialized *Or83bGAL4>UASKir2*.*1* males is comparable (*p >* 0.05) to that of solitary controls (e, right), whereas socialized parental (*Or83bGAL4/+*, *p <* 0.05 and *UASKir2*.*1/+*, *p <* 0.005; *n* = 13–16 per group per genotype) males show a statistically significant increase in sleep as compared to solitary controls. Daytime sleep-enhancement in parental control flies is significantly greater (*p <* 0.0001 for both) than that in silenced flies. Horizontal lines with asterisks above a pair of bars indicate statistically significant difference in sleep-enhanced in the experimental versus control genotypes. Other details are same as in Fig 1.

To examine the role of vision, we used *norpA* mutants, known to have defects in their photo-transduction ability [22]. Socialization had a statistically significant effect on day as well nighttime sleep (*p <* 0.0005 for daytime and *p <* 0.02 for nighttime, *t*-test), which rules out the role of vision in sleep-enhancement. To examine this further, we paired *CS* or *Iso31* males for 4 days under constant darkness (DD). After social interaction, individuals were separated and transferred to LD12:12 at around lights-OFF to record their locomotor activity behaviour. Males from both the strains showed a statistically significant increase (*p* < 0.02 for *CS* and *p* < 0.01 for *Iso31*, *t*-test; Fig 4b and 4c) in daytime sleep, which confirms that vision is not involved in socialization mediated sleep-enhancement.

We probed the role of gustatory signals by examining sleep in socialized flies with ablated gustatory receptor neurons. There are two major groups of gustatory receptor neurons, which respond to either bitter or sweet compounds—*Gr66a* is expressed in the bitter sensitive neurons, whereas *Gr5a* is expressed in the sweet sensitive neurons [23]. Socialized males with ablated bitter sensing neurons (*Gr66aGAL4>UAShid*) sleep significantly more as compared to their solitary counterparts (*p <* 0.0001, *t*-test; Fig 4d). We confirmed the efficiency of ablation by co-expressing Enhanced Green Fluorescent Protein (EGFP) along with *hid* using *Gr66aGAL4* driver and the results suggest a clear reduction of neuronal projections in the fly brain (S1 Fig). Similarly, socialized males with ablated sugar sensing neurons showed a statistically significant sleep-enhancement (*p <* 0.05, *t* test). The gustatory receptors *Gr33a* are known to be involved in male-male interactions [24], and so we asked if *Gr33a* plays any role in sleep-enhancement as a result of pair-wise social interaction in males. Ablated *Gr33a* neurons (*Gr33aGAL4>UASdti*) showed a statistically significant increase in sleep during day as well as nighttime as compared to solitary controls (*p <* 0.0001, *t*-test; Fig 4d). Taken together these results suggest that gustatory receptor neurons are unlikely to be involved in sleep-enhancement due to pair-wise social interaction between males.

To examine the role of olfaction, we used *Orco* null mutant (*Or83b*^0^, henceforth *Orco* flies), known to have compromised ability to sense most odors [25]. Socialization in *Orco* flies did not lead to any statistically significant change in sleep (*p* = 0.93 by ANOVA). Post hoc multiple comparisons using Tukey’s test revealed that socialized *Orco* males sleep as much as solitary controls (*p* = 0.99; Fig 4e), whereas socialized *Iso31* males sleep significantly more as compared to solitary controls (*p* < 0.05). Furthermore, daytime sleep-enhancement in *Orco* males is significantly lower than *Iso31* males (*p* < 0.05) suggesting that olfactory cues are involved in sleep-enhancement. To examine this further, we silenced *Orco* neurons by expressing inward rectifier potassium channels (K_ir_) and found that socialized *Orco* silenced (*Or83bGAL4>UASKir2*.*1*) males sleep as much as solitary controls (*p* > 0.05; Fig 4f), whereas socialized parental (*Or83bGAL4/+* and *UASKir2*.*1/+*) males sleep significantly more than solitary controls (*p* < 0.05 for *Or83bGAL4/+* and *p* < 0.005 for *UASKir2*.*1/+*; Fig 4f). Furthermore, daytime sleep-enhancement in parental control males was significantly greater (*p* < 0.0001) than that of flies where *Orco* neurons were electrically silenced. These results suggest that olfactory signaling mediates sleep-enhancement due to pair-wise social interaction in males.

### Sleep-enhancement does not require circadian clocks

**Fig 5:**
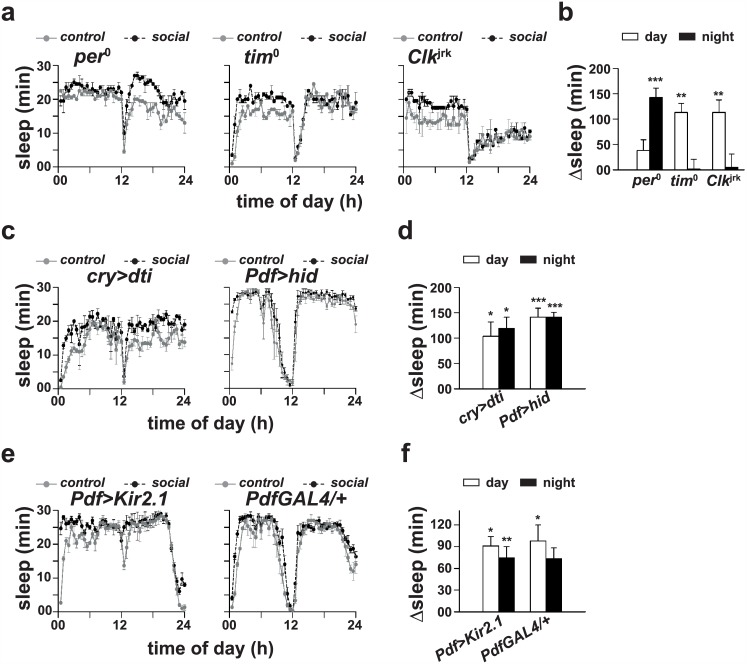
Sleep-enhancement does not require core circadian clock genes and circadian clock neurons. Sleep profiles of (a, left) *per*^0^, (a, middle) *tim*^0^ and (a, right) *Clk*^jrk^ after 4 days of social interaction. (b) Sleep analysis revealed that daytime sleep of socialized *tim*^0^ and *Clk*^jrk^ males is significantly greater as compared to solitary controls. In *per*^0^ flies, although daytime sleep of socialized flies does not show any change, its nighttime sleep is significantly increased as compared to solitary controls (*p <* 0.0005, Student’s *t*-test; *n* = 32 and 19 for socialized and control groups respectively). Change in sleep in *tim*^0^ and *Clk*^jrk^ flies shows a statistically significant increase in daytime sleep (*p* < 0.001 for *tim*^0^ and *p* < 0.005 for *Clk*^jrk^, Student’s *t*-test; *n* = 22 and 17 for socialized and control groups respectively for *tim*^0^, and *n* = 27 and 23 for *Clk*^jrk^) of socialized males from both the genotypes as compared to solitary controls. (c, left) Sleep profiles of *cryGAL4>UASdti* and (c, right) *PdfGAL4>UAShid* males following pair-wise social interaction. (d) Sleep analysis revealed that day as well as nighttime sleep of socialized *cryGAL4>UASdti* males is significantly greater as compared to that of solitary controls (*p* = 0.06 for daytime and *p <* 0.05 for nighttime; *n* = 16 for each group). Also, socialized *PdfGAL4>UAShid* males show an increase in day as well as nighttime sleep (*p <* 0.0005 for both; *n* = 17 and 13 for socialized and control groups respectively) as compared to solitary controls. Sleep profiles of (e, left) *PdfGAL4>UASKir2*.*1* males and (e, right) *PdfGAL4*/+ males following 4 days of pair-wise social interaction. (f) Sleep analysis showed that day as well as nighttime sleep of socialized *PdfGAL4>UASKir2*.*1* males is significantly greater (*p <* 0.05 for daytime and *p <* 0.005 for nighttime; *n* = 12 and 14 for socialized and control groups respectively) as compared to that of solitary controls. Also, socialized *PdfGAL4*/+ males show a significant increase (*p <* 0.05; *n* = 13 and 14 for socialized and control groups respectively) in daytime sleep as compared to solitary controls. Other details are same as in Fig 1.

Circadian clocks are an important component of sleep regulation apart from the homeostatic system and therefore we chose to examine its role in sleep-enhancement. We used three strains (*per*^0^, *tim*^0^ and *Clk*^Jrk^) of flies carrying loss-of-function mutation in core clock genes, known to be arrhythmic for most circadian behaviours [26], and found that socialized *tim*^0^ and *Clk*^Jrk^ males show a statistically significant increase (*p* < 0.001 for *tim*^0^ and *p* < 0.005 for *Clk*^Jrk^, *t*-test; Fig 5a and 5b) in daytime sleep as compared to solitary controls. Although socialized *per*^0^ males did not show a significant increase in daytime sleep (*p >* 0.05), their nighttime sleep was significantly greater (*p* < 0.0001, *t*-test; Fig 5a and 5b) as compared to solitary controls. This suggests that although circadian clocks may not be involved in sleep-enhancement, *per* gene may be critical in promoting sleep during daytime.

### Sleep-enhancement does not require PDF and CRY neurons

The PDF positive clock neurons, apart from timing circadian behaviours, have also been implicated in sleep [19, 20, 27, 28]. Flies with ablated or silenced PDF neurons are usually poor in anticipating lights-on and their evening activity peak is phase advanced with respect to lights-off [29, 30]. Further under DD, PDF manipulated flies are mostly arrhythmic or display weak rhythmicity in activity/rest behaviour with less than 24 h period [26]. PDF manipulated flies used in our experiment displayed both these features (see S2 Fig), which confirms the efficacy of ablation or silencing of PDF neurons. Socialization brought about statistically significant increase in sleep in PDF manipulated males (*p* < 0.0005 by ANOVA). Post hoc multiple comparisons by Tukey’s test revealed that socialized PDF ablated (*PdfGAL4>UAShid*) males sleep significantly more during day as well as nighttime as compared to solitary controls (*p* < 0.0005; Fig 5c and 5d), which suggests that PDF neurons are not involved in sleep-enhancement. In a separate experiment we blocked signaling from PDF neurons by silencing them using *UASKir2*.*1* (*PdfGAL4>UASKir2*.*1*) and found that socialized PDF silenced males sleep significantly more as compared to solitary controls (*p* < 0.0001 by ANOVA). Post hoc multiple comparisons by Tukey’s test revealed a statistically significant increase in day as well as nighttime sleep in socialized males as compared to solitary controls (*p* < 0.05 for daytime and *p* < 0.005 for nighttime sleep; Fig 5e and 5f), which confirms that PDF neurons are not involved in sleep-enhancement. These results suggest that *Drosophila* has evolved different strategies to respond to social cues depending upon the social context, which is primarily determined by the composition of the social group.

Previous studies have also implicated CRYPTOCHROME (CRY) positive neurons in the regulation of sleep [31]. CRY is expressed in the majority of circadian clock neurons and is considered a key player in the reception of photic signals responsible for synchronizing circadian clocks [26]. Ablation of CRY neurons is known to result in arrhythmic activity/rest behaviour in DD and in compromised morning and evening anticipatory activity under LD12:12 [26, 29, 30]. Socialization in CRY ablated flies had a statistically significant effect on sleep (*p* < 0.005 by ANOVA). Post hoc multiple comparisons using Tukey’s test revealed that socialized CRY ablated males sleep significantly more during day (*p* = 0.06; Fig 5c and 5d) as well as nighttime (*p* < 0.05; Fig 5c and 5d) as compared to solitary controls, suggesting that CRY neurons are not involved in sleep-enhancement. These results suggest that circadian clock neurons are unlikely to be involved in social interaction mediated sleep-enhancement in *Drosophila*, particularly when males interact one-on-one with other males.

### Dopamine signaling mediates sleep-enhancement

**Fig 6:**
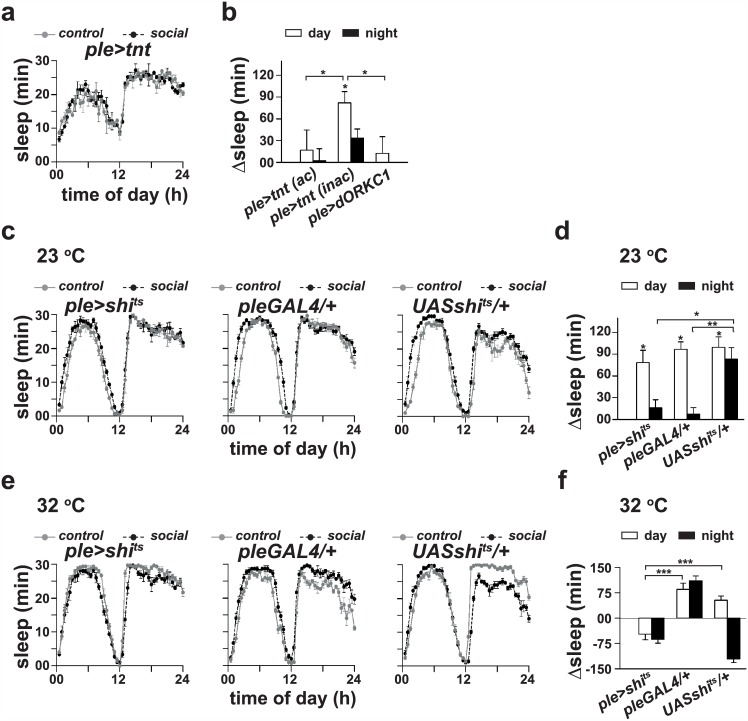
Sleep-enhancement requires dopamine signaling. (a) Sleep profiles of *pleGAL4>UAStnt* (*active*) and (b) change in sleep of *pleGAL4>UAStnt* (*ac-active* and *inac-inactive*) and *pleGAL4>UASdORKC1* males following pair-wise social interaction. (b) Sleep analysis revealed that there is no increase (*p >* 0.05; *n* = 14–20) in daytime sleep in response to social interaction in *pleGAL4>UAStnt(ac)* and *pleGAL4>UASdORKC1* males, whereas sleep is significantly greater in socialized *ple>tnt (inac)* males (*p <* 0.01; *n* = 16 for each group) as compared to solitary controls. Further daytime sleep-enhancement in control flies was significantly greater (*p <* 0.05) than that of silenced flies. Horizontal lines with asterisks above a pair of bars indicate statistically significant difference in sleep-enhanced between the experimental and control genotypes. (c) Sleep profiles and (d) change in sleep of *pleGAL4>UASshi*^*ts*^, *pleGAL4*/+ and *UASshi*^*ts*^/+ males following 4 days of pair-wise social interaction at 23°C. (d) Sleep analysis revealed that increase in daytime sleep (*p* < 0.05; *n* = 16–32) in socialized males is comparable between the three genotypes. (e) Sleep profiles and (f) change in sleep of *pleGAL4>UASshi*^*ts*^, *pleGAL4*/+ and *UASshi*^*ts*^/+ males following 4 days of pair-wise social interaction at 32°C. (f) Sleep analysis revealed that change in sleep due to social interaction is significantly smaller in *pleGAL4>UASshi*^*ts*^ as compared to both the parental controls (*p <* 0.0005 for both *pleGAL4*/+ and *UASshi*^*ts*^/+; *n* = 15–30). Horizontal lines with asterisks above a pair of bars indicate statistically significant difference in sleep-enhanced between the experimental and control genotypes. Other details are same as in Fig 1.

Dopamine has also been implicated in sleep-enhancement in *Drosophila* [18]. When we silenced dopaminergic neurons by expressing active form of tetanus toxin (*tnt*—active), we found that socialized males sleep as much as solitary controls (*p* > 0.05, ANOVA followed by post hoc multiple comparisons by Tukey’s test), whereas socialized males with the inactive form of *UAStnt* (inactive) sleep significantly more during daytime (*p* < 0.01; Fig 6a and 6b) as compared to solitary controls. These results suggest the role of dopamine signaling in sleep-enhancement. In a separate experiment we electrically silenced dopaminergic neurons by expressing an open rectifier Potassium channel *dORKC1* and found that sleep levels of socialized dopaminergic neuron silenced males were comparable to that of solitary controls (*p* > 0.05, ANOVA followed by post hoc multiple comparisons by Tukey’s test; Fig 6b). Daytime sleep-enhancement in controls was significantly greater (*p* < 0.05) than that of silenced flies. To examine the role of dopamine further, we temporally silenced dopaminergic neurons using *UASshibire*^*ts*^ only for the duration of social interaction. When social interaction was carried out at a permissive temperature of 23°C, we observed a statistically significant increase in daytime sleep in socialized *pleGAL4/+*, *UASshibire*^*ts*^/+ and *pleGAL4>UASshibire*^*ts*^ males as compared to their solitary controls (*p* < 0.05, ANOVA followed by post hoc multiple comparisons by Tukey’s test; Fig 6c). However, when social interaction occurred at a restrictive temperature of 32°C, which is known to block synaptic communication due to dysfunctional *Shibire* protein [32], daytime sleep of socialized *pleGAL4>UASshibire*^*ts*^ males was comparable to that of solitary controls (*p* > 0.05), while that of socialized *pleGAL4/+* and *UASshibire*^*ts*^*/+* males was significantly greater than solitary controls (*p* < 0.0005, ANOVA followed by post hoc multiple comparisons by Tukey’s test; Fig 6d). Surprisingly, we also noticed a decrease in nighttime sleep in socialized *pleGAL4>UASshibire*^*ts*^ flies which could probably be due to temperature shift from 32°C to 23°C, since a similar decrease in nighttime sleep was also seen in *UASshibire*^*ts*^ flies. Nonetheless, taken together our results suggest the role of dopamine signaling in sleep-enhancement due to pair-wise social interaction in males.

## Discussion

Fruit fly *D*. *melanogaster* males show a significant increase in daytime sleep and in their tendency to fall asleep sooner, following 3 or more days of pair-wise social interaction with other males. Sleep-enhancement is consistently seen in socialized males from three different “wild type” strains of *D*. *melanogaster* (*CS*, *OR* and *Iso31*), which highlights its ubiquitous nature. It is unlikely that transfer of flies from 7 mm tubes to 5 mm tubes may have caused sleep-enhancement in the socialized flies because sleep-enhancement is estimated relative to solitary individuals, which are also subjected to a similar transfer of tubes. Moreover, in a previous study in *Drosophila* it was shown that size of the tube (2 cc tube *vs* 40 cc vial) does not have any measurable effect on sleep levels [18]. Interestingly, we observed only a marginal effect of socialization on sleep in males that interacted just for one day (Fig 1), which prompted us to consider that sleep-enhancement observed in flies after 4 days of socialization may partly be contributed by sleep loss during the interaction. However, we did not detect any sleep loss in flies while they were interacting socially (Fig 2). Additionally, if this phenomenon was because of a constraint in space, females, which are larger in size, when maintained under similar conditions would suffer a greater sleep loss during the interaction, and hence would show a pronounced sleep-enhancement. However, on the contrary, socialized females do not show any sleep-enhancement. Therefore, sleep-enhancement in males following 4 days of pair-wise social interaction is due to socialization and not due to increased wakefulness.

This sleep phenotype is restricted only to males in spite of the fact that during daytime males sleep significantly more than females [32, 33]. This could possibly be because social cues are weaker when flies interact in pairs as compared to when they interact in groups. Moreover, it is known that the extent of sleep-enhancement due to socialization is a function of group size [18]. Therefore, it is possible that females require stronger social cues to exhibit sleep-enhancement as compared to males. Sleep-enhancement due to group-wise social interaction among males and/or females is known to be mediated by olfactory and visual cues, whereas sleep-enhancement resulting from pair-wise social interaction in males is mediated via olfaction alone, while visual and other sensory modalities are dispensable, which further highlights the uniqueness of this sleep phenotype. This is not surprising because olfactory cues are known to induce neuronal plasticity and play a vital role in mediating key behaviours such as learning and memory and social interactions [34, 35].

It is believed that during the early phase of social interaction, sensory cues cause activation of l-LN_v_ neurons, which results in increased synaptic terminals in l-LN_v_ neurons [20]. During the later phase of social interaction, homeostatic mechanisms come into play, which decrease l-LN_v_ excitability resulting in sleep-enhancement [20]. Sleep-enhancement resulting due to pair-wise social interaction between males does not require functional circadian clocks, which suggests that it is primarily driven by homeostatic processes. Furthermore, loss of sleep during the interaction was comparable to that of solitary controls which suggests that sleep-enhancement resulting from pair-wise social interaction is not caused due to previous wakefulness and/or l-LN_v_ excitation. Interestingly, previous studies have suggested wake promoting l-LNv neurons to play a critical role in sleep-enhancement, whereas we found that both ablation as well as silencing of PDF neurons does not have any detectable impact on sleep-enhancement, which rules out the role of PDF neurons in sleep-enhancement resulting due to pair-wise social interaction between males. In addition, flies with ablated CRY neurons, of which PDF neurons are a part, continue to show sleep-enhancement, thus confirming that circadian clock neurons are not involved in socialization mediated sleep-enhancement. As discussed previously, such differences in the role of circadian clock neurons could probably be due to the fact that social environments in previous studies were highly enriched due to group-wise interaction among 30–40 flies of both sexes, while in the present study interaction occurred between two males only.

In a previous study it was shown that the gene *rutabaga*, which encodes adenylate cyclase, plays an important role in sleep-enhancement [18]. We found that socialized *rutabaga* mutant males do not show sleep-enhancement (data not shown), which suggest that *rutabaga* is involved in sleep-enhancement caused due to pair-wise social interaction between males. Another key signaling molecule, dopamine, has been reported to be crucial for sleep-enhancement resulting due to group-wise social interaction [18]. In consensus with this finding, we also found that dopamine signaling is necessary for sleep-enhancement due to pair-wise social interaction because socialized males with manipulated dopaminergic neurons do not show sleep-enhancement. Thus, it appears that in *Drosophila*, although neuronal responses to social cues may vary, the basic mechanisms underlying socialization mediated sleep-enhancement appears to be conserved across social contexts.

## Conclusions

*Drosophila* appears to have evolved different strategies to respond to social cues in a context dependent manner. While ‘waking experience’ soon after emergence is critical for eliciting sleep-enhancement in a group-wise social interaction, socialization alone is necessary and sufficient to cause sleep-enhancement in a pair-wise social interaction. We have thus defined the minimal conditions in which *Drosophila* males respond to social cues eliciting sleep-enhancement, and have shown that this sleep phenotype is male-specific and is not an immediate rebound of sleep loss during the social interaction. Socialization mediated sleep-enhancement in males requires dopamine signaling and is mediated by olfaction, but does not involve circadian clocks.

## Supporting Information

S1 Fig Verification of Gr66aGAL4 and UAShid flies. Representative adult brains showing (left) *Gr66aGAL4* driven *UAS2XeGFP* expression stained with antibody against GFP. Several neuronal projections (arrows) in the Suboesophageal Ganglion (SOG) region are seen just under the Posterior Optic Tract (POT) area. (right) *Gr66aGAL4* driven *hid* ablates the neurons which results in decreased projections in the SOG as marked by the dotted box. Asterisks indicate artifact. Scale bars are 50 μm. Dissected brains of both genotypes were first fixed with 4% Paraformaldehyde and then blocked with 10% horse serum. Brains were flooded with primary antibody against GFP (anti-GFP, chicken, 1:2000) and later after 3–4 washes with 0.5% phosphate buffer Triton-X, with secondary antibody (anti-chicken-Alexa 488, 1:3000) for an hour each. Brains were mounted on slides in 7:3 PBS: Glycerol medium and were imaged using an epifluorescence microscope (Zeiss Axio Observer Z1). Brains (*n* = 4) were sampled for each genotype. For the experimental genotype where *UAShid* was co-expressed with *UASGFP*, in 3 brains no GFP was detectable in the central brain whereas in one case, very few projections from the Gr66a neurons were visible in the posterior central brain area.
(EPS)

S2 Fig Verification of pdfGAL4, UASKir2.1 and UAShid flies. (a) Representative actograms for flies in which pigment dispersing factor (PDF) positive neurons have been ablated (*UAShid*) or silenced (*UASKir2*.*1*) in constant darkness (DD) show the expected phenotype of arrhythmicity. (b) Activity/rest profiles of *PdfGAL4>UAShid* and *PdfGAL4>UASKir2*.*1* flies in 12:12 h light/dark cycles (LD12:12) shows the expected phenotype of reduced morning anticipation and advanced evening peak. (c) Bar diagram showing percentage arrhythmicity in *PdfGAL4>UAShid* and *PdfGAL4>UASKir2*.*1* males. We found around 67% of *PdfGAL4>UAShid* males and around 89% of *PdfGAL4>UASKir2*.*1* males to be arrhythmic under DD.
(EPS)
